# The Role of D-3-Phosphoglycerate Dehydrogenase in Cancer

**DOI:** 10.7150/ijbs.41051

**Published:** 2020-03-05

**Authors:** Xiaoya Zhao, Jianfei Fu, Jinlin Du, Wenxia Xu

**Affiliations:** 1 Central Laboratory, Jinhua Hospital of Zhejiang University, Jinhua 321000, Zhejiang Province, China; 2 Department of Medical Oncology, Jinhua Hospital of Zhejiang University, Jinhua 321000, Zhejiang Province, China; 3 Department of Colorectal Surgery, Jinhua Hospital of Zhejiang University, Jinhua 321000, Zhejiang Province, China

**Keywords:** serine metabolism, SSP, structure of PHGDH, expression of PHGDH, cancer, non-metabolic activity, inhibitor of PHGDH, tumor resistance

## Abstract

Serine, a non-essential amino acid, can be imported from the extracellular environment by transporters and *de novo* synthesized from glycolytic 3-phosphoglycerate (3-PG) in the serine biosynthetic pathway (SSP). It has been reported that active serine synthesis might be needed for the synthesis of proteins, lipids, and nucleotides and the balance of folate metabolism and redox homeostasis, which are necessary for cancer cell proliferation. Human D-3-phosphoglycerate dehydrogenase (PHGDH), the first and only rate-limiting enzyme in the *de novo* serine biosynthetic pathway, catalyzes the oxidation of 3-PG derived from glycolysis to 3-phosphohydroxypyruvate (3-PHP). PHGDH is highly expressed in tumors as a result of amplification, transcription, or its degradation and stability alteration, which dysregulates the serine biosynthesis pathway via metabolic enzyme activity to nourish tumors. And some recent researches reported that PHGDH promoted some tumors growth via non-metabolic way by upregulating target cancer-promoting genes. In this article, we reviewed the type, structure, expression and inhibitors of PHGDH, as well as the role it plays in cancer and tumor resistance to chemotherapy.

## Introduction

Metabolic reprogramming plays an important role in the development of tumors 1. Glucose is one of the most primary nutrients utilized for mitochondrial oxidative phosphorylation to generate energy supporting cellular behaviors in normal differentiated cells. In contrast, most cancer cells exploit glucose for aerobic glycolysis to support the proliferation of cancer cells 2, which is called the “Warburg effect” 3. Despite the universal alterations in glucose metabolism observed in tumors 2-4, diversion of glycolytic flux into amino acid synthesis is also necessary during tumor development 5, 6. Serine, an unessential amino acid, is required for certain anabolic processes 7. It is a critically important component of proteins and provides precursors for the synthesis of lipids, such as sphingolipids and phosphatidylserine (PS) 8, a major component of cellular membranes 7, 9. Serine is a vital source of “one-carbon” units carried by tetrahydrofolate supplying methyl groups for the modification of lipids, nucleic acids, and proteins 9. Thus, serine serves multiple essential roles in cellular metabolism.

**Figure 1:**
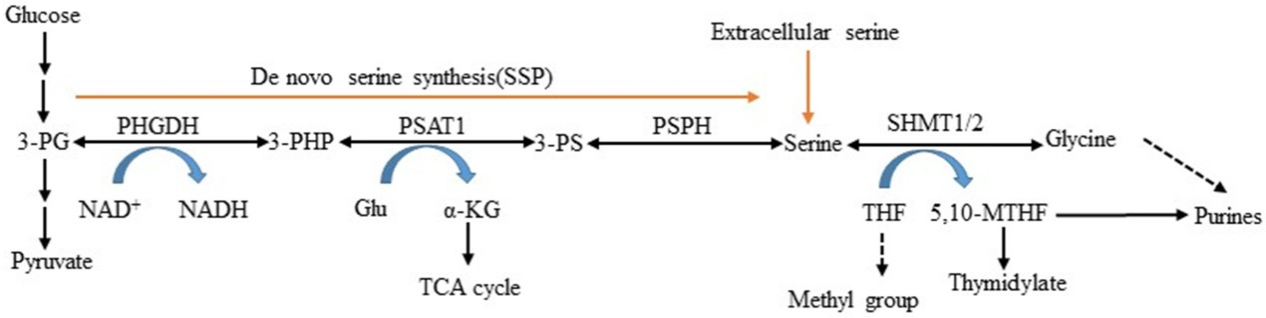
** L-serine synthesis pathway.** PHGDH first catalyzes the oxidation of 3-phosphoglycerate (3-PG) to 3-phosphohydroxypyruvate (3-PHP), with the coinstantaneous reduction of the cofactor NAD^+^ to NADH. The subsequent transamination reaction is catalyzed by phosphoserine aminotransferase (PSAT), which uses glutamate (Glu) as a nitrogen donor and thereby converts 3-phosphoserine (3-PS) and α-ketoglutarate (α-KG) into tricarboxylic acid (TCA) cycle. Dephosphorylation of phosphoserine via phosphoserine phosphatase (PSPH) produces serine, and then serine hydroxymethyltransferase (SHMT) converts serine into glycine and 5,10-methylenetetrahydrofolate (5,10-MTHF) via tetrahydrofolate (THF) supplying methyl.

Serine is obtained exogenously via amino acid transporters and also can be synthesized from a branch of glycolysis 5. NAD^+^-dependent enzyme 3-phosphoglycerate dehydrogenase (PHGDH) is the first and only rate-limiting enzyme in the serine and glycine *de novo* biosynthetic pathway 10. It catalyzes glycolytic intermediate 3-phosphoglycerate (3-PG) transformed into 3-phosphohydroxypyruvate (3- PHP) 11, 12. Subsequent enzymatic reactions in the pathway convert 3-PHP to serine via transamination (PSAT1) and phosphate ester hydrolysis (PSPH). Serine can be converted to glycine by serine hydroxymethyltransferase (SHMT) and then synthesize glutathione (GSH) to protect against oxidative stress 13, 14. This process involves two important factors: PSAT1 converts glutamate to alpha-ketoglutarate (α- KG) serving as fuels for the TCA cycle, and SHMT1/2 catalyzes serine that is cleaved into CH_2_-THF in either the cytosol or mitochondria, which is necessary for the biosynthesis of thymidine and purine (Figure 1). In this study, we reviewed PHGDH's structures and functions, described its role in serine metabolism, and explored its association with diseases.

## The expression of 3-phosphoglycerate dehydrogenase

The nucleotide sequence of human PHGDH gene located at 1p12, which has 533 amino acid open reading frames (ORFs) sharing 88% and 94.0% homology with those of rat and mouse PHGDH, respectively 15, encodes a 56.6 kDa protein. However, the sequence similarity of the proximal promoter region (700 bp) of human PHGDH is 42% and 40% identical to its rat and mouse counterparts, respectively 16, 17. This means that the regulation mechanism of human PHGDH gene expression differs from that of rats and mice. Similar to mouse PHGDH promoter, which previously demonstrated multiple transcription initiation sites at -136, -83, -81, -79, and -74 bases upstream from the first ATG codon, the human PHGDH promoter has Sp1 and NF-Y- binding sites in the absence of a TATA-box motif and thus showed multiplicity of transcriptional initiation sites 18, 19. Two different transcripts of 3-PHGDH mRNA were detected in normal human tissues. A primary 2.1 kb transcript was markedly expressed in the testes, kidneys, ovaries, prostate, brain, liver, and pancreas and expressed at low levels in the colon, thymus, and heart. A 710bp transcript was also found at low levels predominantly in the heart and skeletal muscle 15. Gromova et al. discovered that PHGDH has two major protein variants called α and β differing by approximately 3kDa in size and with each showing multiple modifications 20. However, they did not prove that their sizes were consistent with the expected sizes of ENSEMBL transcripts ENST00000369409 and ENST00000369407 of PHGDH. Both translate into 533aa and 499aa proteins at 53.1 kDa and 56.6 kDa, respectively. Gromova et al. also found that the expression of PHGDH β variants could be caused by malignancy or deterioration of a malignant phenotype, but their relationship is unclear.

## The type and structure of 3-phosphoglycerate dehydrogenase

**Figure 2:**
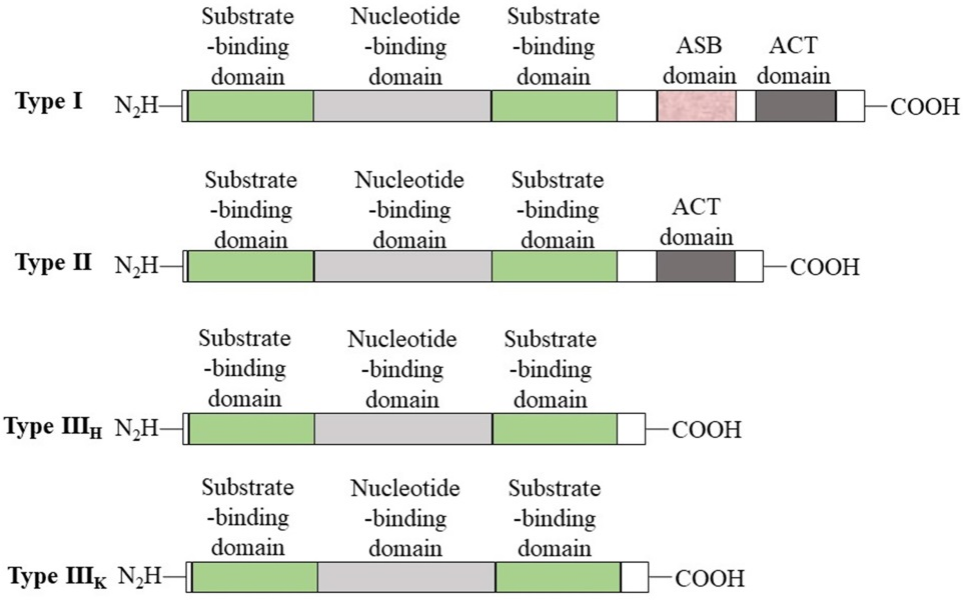
** Basic domain structure of PHGDH.** Basic domain structure found within the three enzyme types of PHGDH. Additional amino acids at the N-terminus change depending on the species. Two forms of the type III enzyme exist depending on whether histidine (type H) or lysine (type K) is present at the active site.

**Figure 3:**
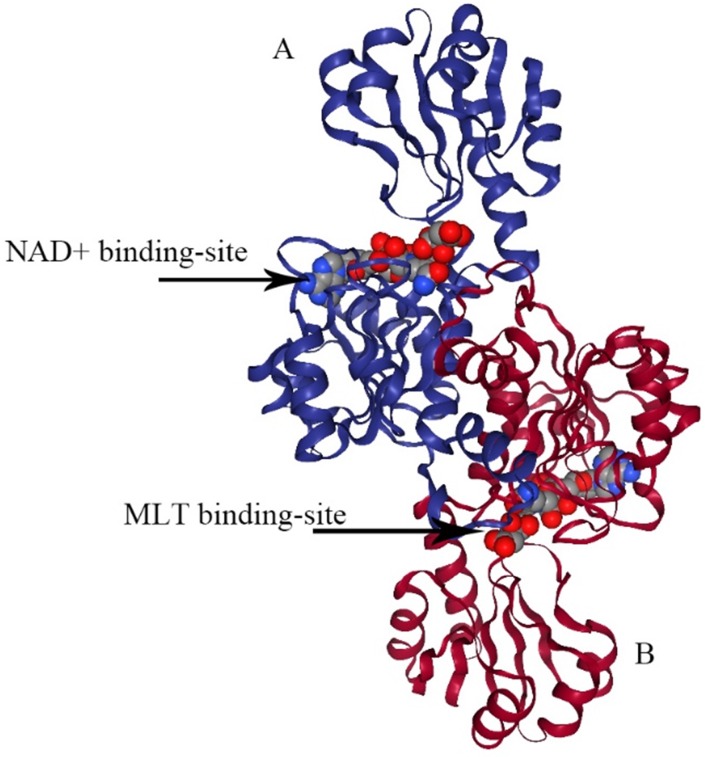
** The structure of type III PHGDH.** Each subunit is colored differently. NAD+ and D-malate (MLT) binding-site are designated with arrows.

PHGDH is universally expressed in all organisms in at least three different basic structural forms, called types I, II, and III (Figure 2) 21. These forms do not appear to be strictly specific for organism type, such as human, *M. tuberculosis.* rat and synechocystis have similar type I structures. PHGDH type III contains only substrate-binding and nucleotide-binding domains, which are structurally distinct and joined by two polypeptide chain segments at the active cleft site (Figure 3). *Pyrococcus, Rhodopseudomonas* and *Clostridium* have similar type III H, while *Entamoeba hystolytica, Baterioides fragilis* and *Porphyromonas gingivalis* have similar type III K. Type II has an aspartate kinase-chorismate mutase- tyrA perrhenate dehydrogenase (ACT) domain, a regulatory domain consisting of approximately 60-70 amino residues, and a βαββαβ structure. The ACT domain has been reported to function as a binding site for L-serine to provide feedback inhibition in *E. coli* and *M. tuberculosis.* However, this regulatory mechanism could not be confirmed for human PHGDH 22, 23. The type I enzyme has an additional regulatory domain at the carboxyl terminal extremity, allosteric substrate-binding (ASB) domain, composed of approximately 150 amino acid residues with an αβααββ motif. The ASB domain is found between the substrate-binding domain and ACT domain and has been studied only in *M. tuberculosis* 24. The function of the ASB domain is to provide additional levels of allosteric control. To date, no other protein containing the ASB domain has been found, except for L-serine dehydratase from *Legionella pneumophila* 25.

**Figure 4:**
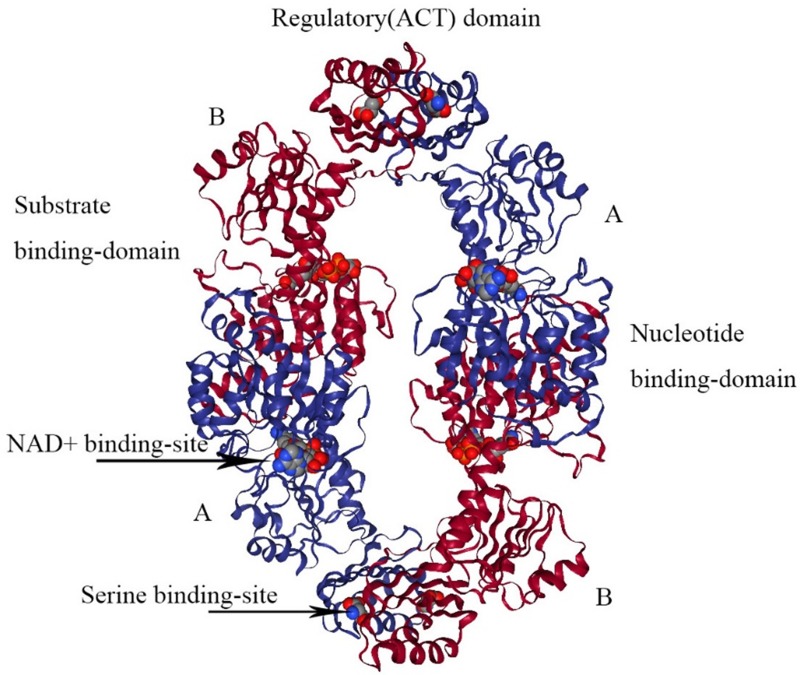
** The structure of *E. coli* PHGDH.** The tetrameric structure of *E. coli* PHGDH is shown with NADH bound at the active sites and L-serine bound at the effector sites, which are designated with arrows. The subunits (A and B) are different colors for clarity and the structural domains of one subunit are indicated. Each subunit has the same amino acid sequence.

*E.coli* PHGDH is a tetramer consisting of selfsame subunits in regard to amino acid sequences. It contains three different structural domains as shown in Figure 4. The crystal structure of* E.coli* PHGDH was discovered by binding NAD+ plus a substrate analog (a-ketoglutarate) to the active site 26 and binding L-serine and NADH to the effector site and catalytic site, respectively 27. When L-serine binds to the effector site, the conformation of the active site changes, resulting in the loss of activity. Binding research clarified that all four sites of the two ACT domains can be occupied with L-serine via a bridge formed by hydrogen bonds. The binding of serine to one site causes the other site to open and the last two sites have a negative cooperative relationship with the binding of the crystal structure 22.

**Figure 5:**
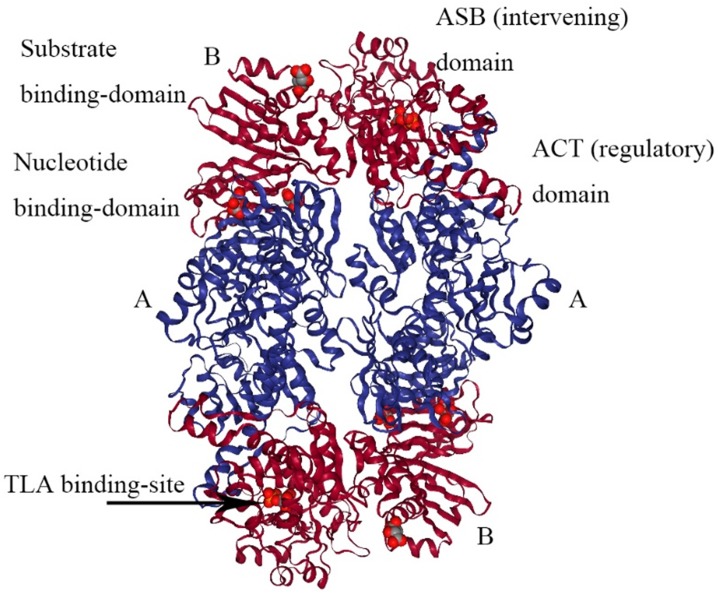
** The structure of *M. tuberculosis* PHGDH.** The structure of *M. tuberculosis* PHGDH (PDB code: 1YGY) is shown with each subunit (A and B) depicted in a different color for clarity. The active site histidine residue is shown with arrows.

The crystal structure of *M. tuberculosis* PHGDH (PDB 1YGY) is dramatically different from that of *E.coli* PHGDH, although the catalytic domains adopt the same conformation in all four subunits. The structure of PHGDH has a distinct asymmetry as a result of the existence of the fourth domain, the ASB domain (Figure 5) 28. To date, the full-length crystal structure of mammalian PHGDH remains unknown. Only the catalytic subunit of human PHGDH (PDB 2G76) has been solved as a dimer, probably because the crystallized truncated protein lacks the regulatory domains that influence inter-subunit interfaces. In human PHGDH, the active site consists of the substrate-binding domain and nucleotide domain. It is lined by several loop regions of the first monomers Arg54-Val59, Ala76-Val83, Asn97-Gly101, Gly152- Leu153, Asp175-Ile178, His206-Leu216, Cys234- Val240, Asp260-Asp269, and Cys281-Ser287 and one loop of the second monomer Trp133-Lys136 29.

## PHGDH expression and cancer

The link between cancer and serine biosynthesis was first suggested by observations that PHGDH activity was greater in rat hepatoma cell lines than in normal liver cells, and the highest PHGDH activity led to the greatest growth rate 30. Subsequent studies showed that PHGDH activity was most consistently increased and correlated with tumor growth in many other tumors 31. To date, it has been reported that the level of PHGDH protein is increased in 16% of all cancers 32, 40% of melanoma samples 4, 70% of estrogen receptor (ER)-negative and triple-negative breast cancer samples, and even associated with subtypes of breast cancers^33^. This renewed interest in the serine synthesis relative with tumor development (Table 1).

In metastatic variants of estrogen receptor-negative breast cancer cells, PHGDH and PSAT protein are highly expressed and related to poor prognosis, decreased overall survival, higher tumor grade, and high expression of the proliferative markers proliferating cell nuclear antigen and Ki-67 33. PHGDH regulates the oxidation of 3-PG, potentially influencing the NAD^+^⁄NADH ratio and cellular redox balance. Certain breast cancers depend on genomically amplified PHGDH, which diverts glucose carbons away from glycolysis into oxidative stress and biosynthetic pathways 4, 34. In gliomas 35 and cervical 36, pancreatic 37, and colorectal cancer 38, the overexpression of PHGDH is associated with advanced TNM stage, large tumor, higher tumor grade, and shorter overall survival time, respectively. In the human pancreatic cancer cell lines BxPC-3 and SW1990, knockdown of PHGDH inhibits the cell proliferation, migration, and invasion abilities by downregulating the expression of cyclin B1, cyclin D1, MMP-2, and MMP-9. In gastric cancer, PHGDH was identified as an independent prognostic factor for outcomes, and the high expression of PHGDH was markedly related to histological type, tumor stage, and preoperative carcinoembryonic antigen 39. Compared to the normal adjacent tissues, PHGDH expression is markedly and significantly elevated in lung adenocarcinoma and positively with poor prognosis. Accordingly, models with high levels of PHGDH display rapid proliferation and migration 10. Reprogramming of tumor metabolism also involves leukemia cells. In leukemia cells (HL-60, K-562, and THP-1), inhibiting Gln metabolism leads to the upregulation of PHGDH, which maintains cell survival. Thus, it is necessary to combine the inhibition of Gln metabolism and serine metabolism in the treatment of hematological malignancies 2.

In most cases, tumor cell proliferation is promoted by increased level of PHGDH and supressed when PHGDH is knocked out or mutated in specific site. 40. In some cases, extracellular serine is sufficient to promote tumor cell proliferation, whereas in other cases, extracellular serine is unable to support cell proliferation when PHGDH is absent. For example, adding excess serine to the growth medium of PHGDH knockdown PANC-1 cells was unable to rescue cell proliferation 41. This suggests that PHGDH can promote cell proliferation not just by providing serine.

## PHGDH plays a non-metabolic role or leads to additional enzymatic activity except supplying serine in cancer

PHGDH knockdown or inhibition inhibits the proliferation of some cancer cells in cultures in a manner that cannot be fully rescued by exogenous serine 4, 33, 42, 43.This demonstrated that PHGDH not only possesses metabolic enzyme activity by supplying serine but also plays a non-metabolic role or leads to additional enzymatic activity in tumorigenesis.

In glioma, PHGDH identified as directly upstream of FOXM1 prevents FOXM1, a proverbial oncogene, from proteasome degradation due to ubiquitination by interacting with its N-terminal, which facilitates tumor proliferation, invasion, and tumorigenicity 35. In the translation initiation process, eIF4E recognizes m7 GTP on mRNA 44 and eIF4A unwinds the 5'mRNA structure to compose pre-initiation complexes 45. In PANC-1 cells, PHGDH was found to interact with the translation initiation factors eIF4A1 and eIF4E and promote the assembly of the complex eIF4F on 5' mRNA structures to contribute to the expression of ZO-1 and E-cadherin involved in tight junctions 41, 46. PHGDH possesses the dinucleotide-binding domain, which is close to its substrate-binding pockets, serving for RNA binding 47, 48. It was speculated that the exact interactions between PHGDH and eIF4A1/eIF4E are related to its dinucleotide-binding domain and might also be close to its substrate-binding pockets. Therefore, reducing PHGDH expression and blocking the binding of PHGDH to eIF4A1/eIF4E provides a new anti-tumor therapeutic design.

PHGDH is a member of the isomer-specific 2- hydroxyacid dehydrogenase family, and its substrate is 3-phosphohydroxypyruvate, which is similar to α-ketoglutarate (α-KG) in structure. Evidence has demonstrated that PHGDH has additional enzymatic activity by converting NADH-dependent reduction of α-KG to the oncometabolite D-2-hydroxyglutarate (D-2HG) in breast cancer 49, 50. D-2HG can competitively restrain α-KG-dependent dioxygenase enzymes, including DNA and histone demethylases 51-55. Large amounts of D-2HG produced by IDH active site mutants in glioma and acute myeloid leukemia (AML) promotes the formation and malignant progression of gliomas and the transformation of leukemic cells 56-58.

The mechanisms of PHGDH amplification promoting tumor growth include supplying serine for protein synthesis and one-carbon metabolism, promoting the TCA cycle, and non-enzymatic functions including FOXM1 binding. Even overproducing the oncometabolite D-2HG impacts cell physiology. In the future, it is essential to explore the functional significance of PHGDH's multiple activities in different physiological and pathological settings.

## The factors controlling PHGDH expression in cancer

PHGDH copy number gain is a reason for PHGDH high expression. PHGDH amplifies more quickly in triple-negative breast cancer than in other breast cancer subtypes, and PHGDH is more highly expressed in estrogen receptor-negative tumors than estrogen receptor-positive tumors 33. PHGDH gene copy number gain is observed at a higher frequency in melanoma than other cancers 4, 33 and PHGDH expression can accelerate melanoma progression in mice 59.

In addition to amplification, PHGDH expression can be upregulated transcriptionally by Sp1/NF-Y 17, activating transcription factor 4 (ATF4)^60^, protooncogene c-Myc^61^, and TGF-β^62^ and epigenetically by lysine methyltransferase G9A^63^. It has been clarified that Sp1 binds to the proximal GC-motif (-194 to -185) and NF-Y binds to the CCAAT-motif (165 to -154), dramatically regulating the promoter activity of the human PHGDH gene in HeLa cells by a ChIP assay. However, the positive regulatory region necessary for transcription was contained within the -196/+4 proximal sequence of the mouse PHGDH promoter, in which one Sp1-binding site was critical for the promoter activity. ATF4 has been reported as both a direct transcriptional target 64, 65 and heterodimerization partner of transcription factor nuclear factor erythroid-2- related factor 2 (NRF2). Human non-small cell lung cancer with high NRF2 protein expression displayed significantly higher expression of ATF4, PHGDH, PSAT1, and SHMT2, which was significantly related to poorer prognosis and higher tumor grade. It has also been verified that in NSCLC, NRF2 induced the expression of PHGDH, PSAT1, and SHMT2 by regulating ATF4 transcriptional activity to support glutathione and nucleotide production through serine biosynthetic metabolism. The indirect regulation mechanisms of ATF4 transcriptional activity by NRF2 are unknown, and it is speculated that NRF2 regulates serine biosynthesis genes combinatorially through ATF4 and additional factors 66. Ben-Sahra reported that ATF4 can be activated by mTORC1 independent of its canonical induction downstream of eIF2α phosphorylation, stimulating nucleic acid synthesis, and helping cells adapt to amino acid deprivation and endoplasmic reticulum (ER) stress 67. Methyltransferases play an essential role in the regulation of transcription by controlling the methylation of histone and DNA. G9A, an H3K9 methyltransferase, has a central role in catalyzing transcriptional activation of the serine-glycine biosynthetic pathway by upregulating the expression of PHGDH, PSAT1, PSPH, and SHMT with H3K9me1, an epigenetic marker associated with active chromatin 68, 69. Moreover, higher G9A expression significantly increased serine and glycine biosynthesis in the cells depending on the upregulation of ATF4 63. In lung adenocarcinomas, differential PHGDH protein levels were defined by their degradation and stability, which were regulated by deubiquitinating enzyme JOSD2 10.

## The inhibitor of PHGDH against cancer

It is critical to find the inhibitor of PHGDH to treat patients with high PHGDH expression or PHGDH gene amplification. Many recent studies have reported compounds with activities against PHGDH.

### Orthosteric inhibitors

**Figure 6:**
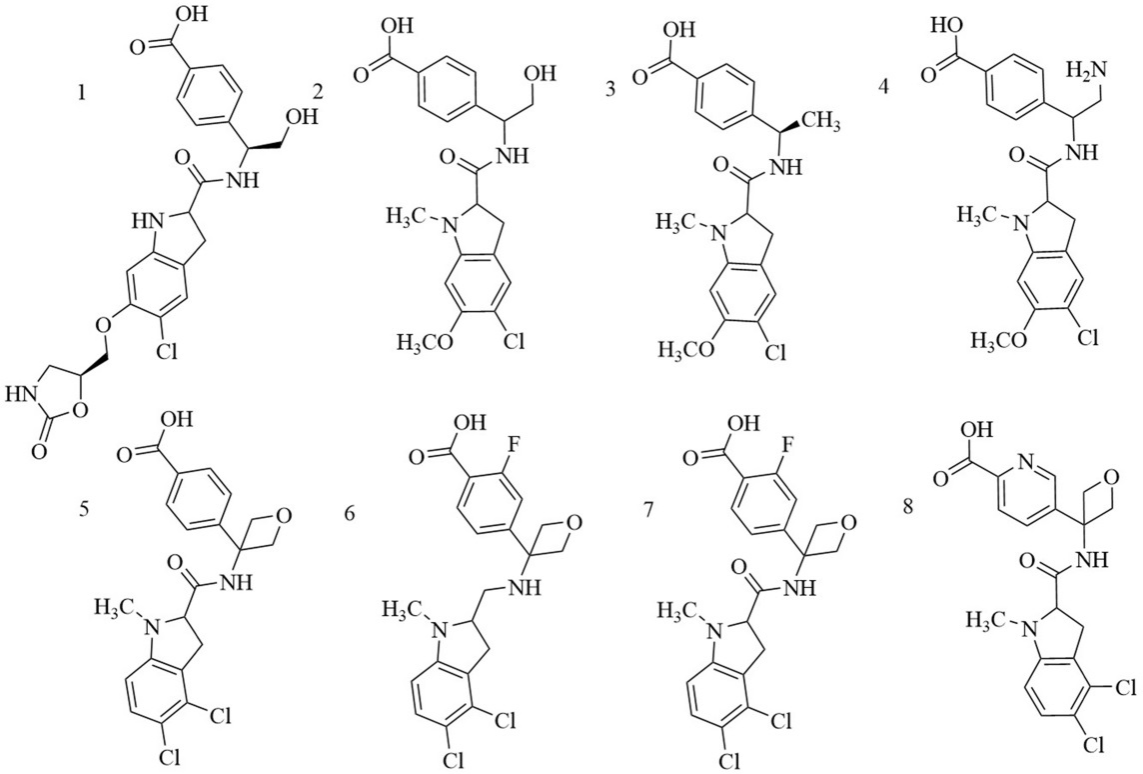

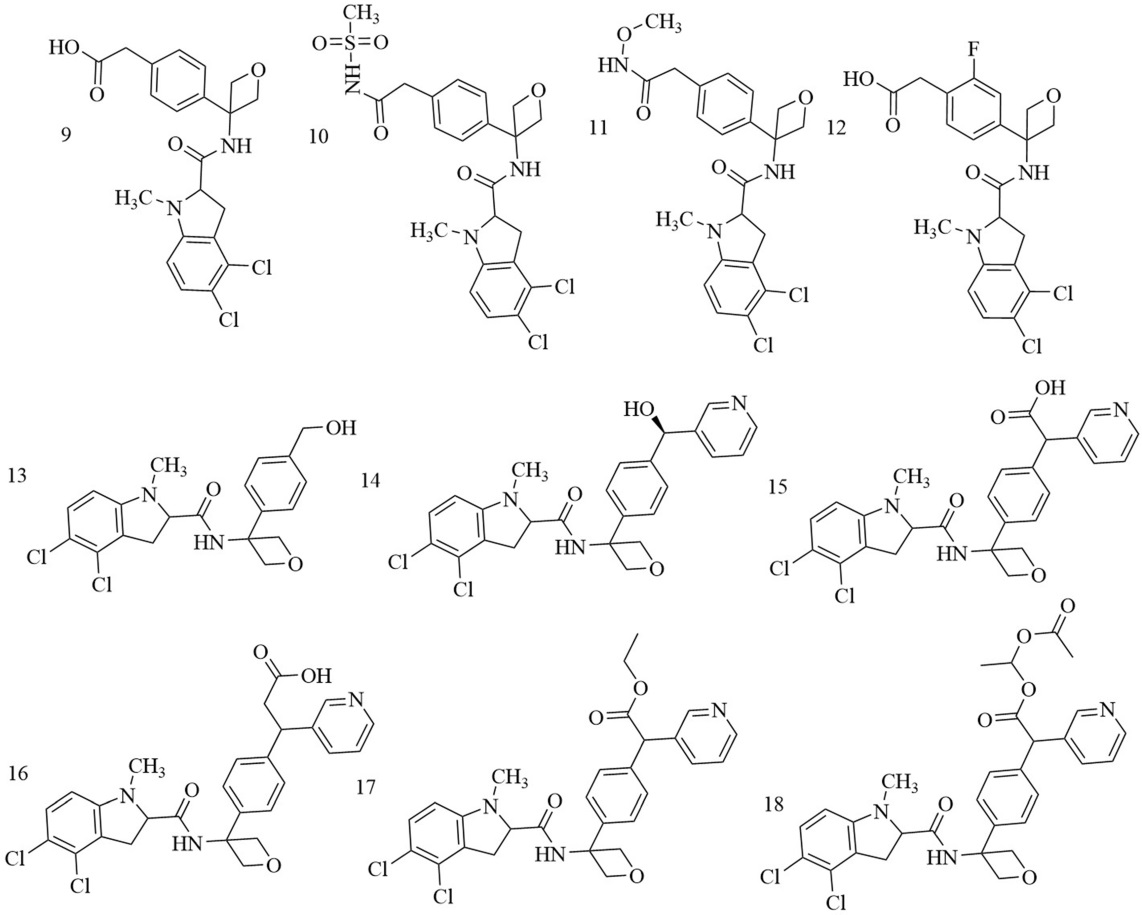
Chemical structures of indole derivatives.

**Figure 7:**
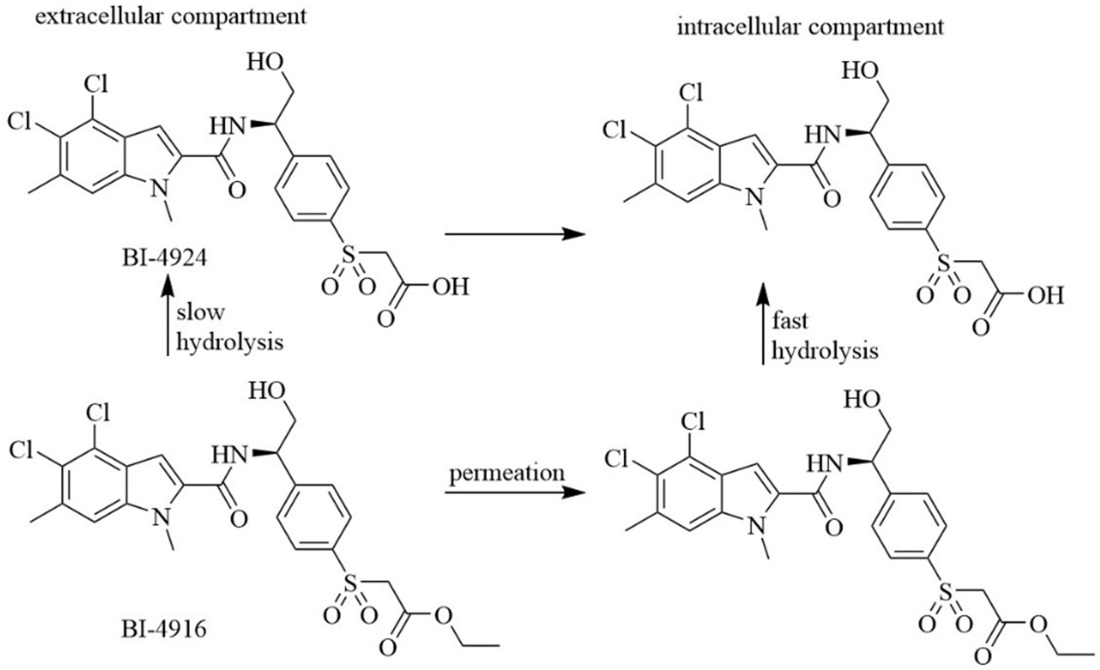
BI-4916, a prodrug of the cofactor nicotinamide adenine dinucleotide (NADH/NAD^+^)-competitive PHGDH inhibitor BI-4924, has shown high selectivity against the majority of other dehydrogenase targets. An intracellular ester cleavage mechanism of the ester prodrug was utilized to achieve intracellular enrichment of the actual carboxylic acid-based drug to overcome high cytosolic levels of the competitive cofactors NADH/NAD^+^.

*Indole derivatives.* Mullarky et al. found a series of that bind the NAD^+^ pocket of PHGDH (Figure 6), inhibiting its activity and arresting the proliferation of cancer cells in serine-free media with low nanomolar affinities 70. Weinstabl discovered a cofactor nicotinamide adenine dinucleotide (NADH/NAD^+^)- competitive PHGDH inhibitor, BI-4916, a prodrug of BI-4942. BI-4916 has shown high selectivity against the high cytosolic levels of the competitive cofactors NADH/NAD^+^ with high selectivity via an intracellular ester cleavage mechanism of the ester prodrug to achieve intracellular enrichment of the actual carboxylic acid-based drug (Figure 7) 71.

### Allosteric inhibitors

**Figure 8:**
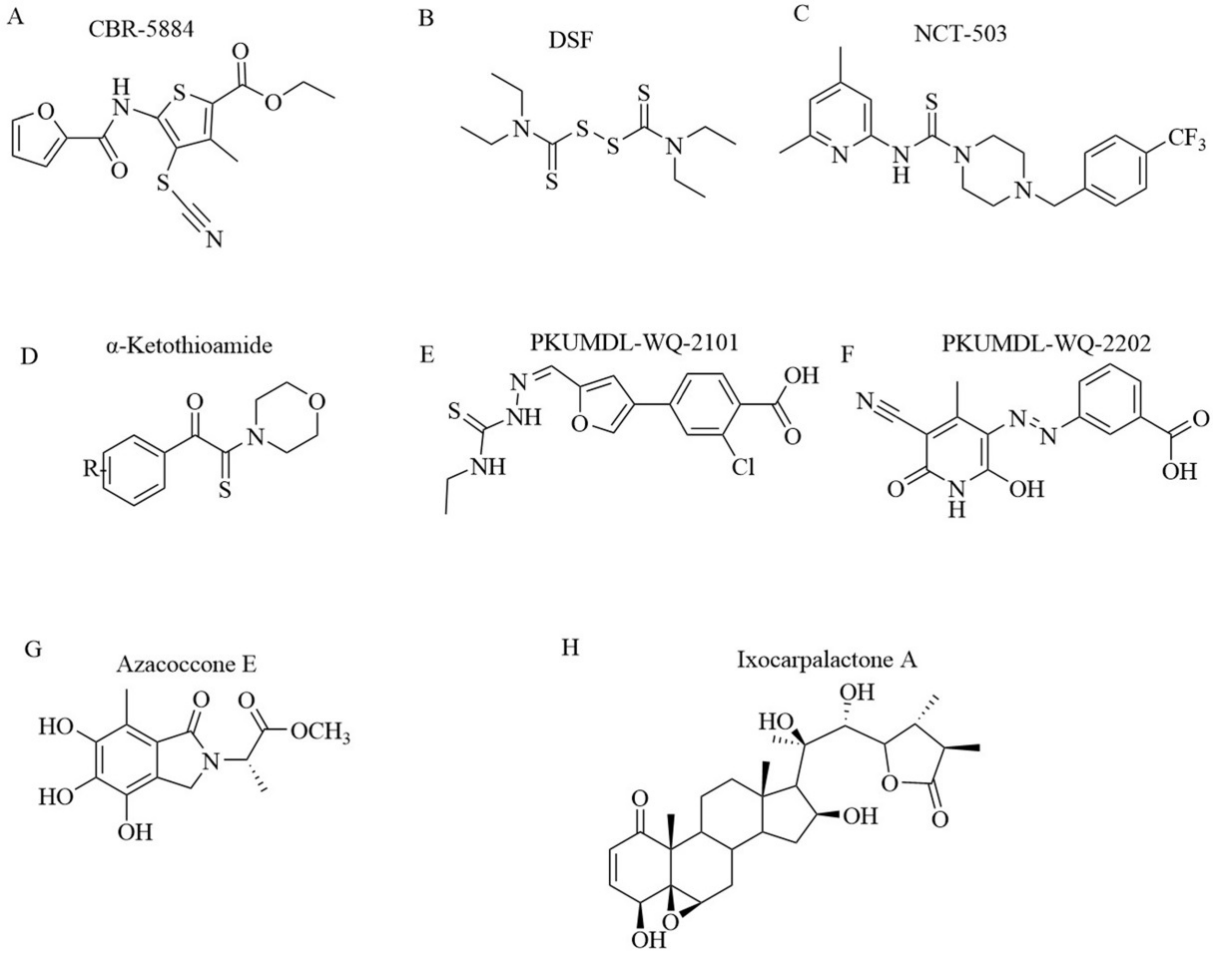
** Chemical structures of PHGDH inhibitors.** (A) CBR-5884. (B) DSF. (C) NCT-503. (D)*α*-ketothioamides. (E) PKUMDL-WQ-2101. (F) PKUMDL-WQ-2202. (G) Azacoccone E. (H) Ixocarpalactone A.

Mullarky et al. discovered PHGDH inhibitor by screening a library of 800,000 drug-like compounds and identified Compound 9 (CBR-5884) (Figure 8A) as superior 42. CBR-5884 inhibited PHGDH enzymatic activity in a time-dependent manner with an IC50 of 33 ± 12 μM. CBR-5884 was identified as a covalent inhibitor that binds to a Cys in the non-active site and disrupts its oligomeric state. CBR-5884 inhibited the growth of cells by 35% to 60% in serine-abundant media, and by 80% to 90% in serine-depleted media at 30 μM. The substrate-binding pocket of PHGDH is rather small, approximately 100-200 Å3, and the physiological concentration of its cofactor NAD^+^ is as high as 0.3 mM 72. These properties likely increase the difficulties of designing substrate-competitive inhibitors. Because NAD+ or NADH is a widely used cofactor, many studies focused on designing allosteric inhibitors for PHGDH that do not compete with the native ligand. Allosteric regulation can be achieved by various effectors, ranging from micromolecules to macromolecules and have high specificity, as allosteric-binding sites are not usually evolutionarily conserved 73. Neither a direct binding test nor a postulated binding site was reported. CBR-5884 was unstable in mouse plasma and could not be used for *in vivo* testing. Spillier et al. found that disulfiram (DSF) (Figure 8B), an anti-alcohol drug, also inhibited PHGDH activity via converting PHGDH tetramer into either an inactive dimer covalently linked by a disulfide bridge involving Cys116 on adjacent monomers or, to a lesser extent, an inactive intermediate^74^.

Similar to Mullarky et al., Pacold et al. found three PHGDH inhibitors by first screening a 400,000-compound NIH Molecular Libraries Small Molecule Repository (MLSMR) library and then optimizing the lead compounds 75. The best compound, NCT-503 (Figure 8C), exhibited an IC50 value of 2.5 ± 0.6 μM and showed some selectivity in PHGDH-amplified breast cancer cell lines and had bioactivities in a xenograft model. NCT-503, also a non-competitive inhibitor in regards to substrates and cofactors, closely binds to the active site as a mutation of C234 in the protein's active site to reduce the inhibitory effect of PHGDH.

*α-ketothioamide derivatives.* Primary screening was implemented on a compound library of 336 molecules from a fragment library and an in-house compound collection at a high concentration (100 μM). Of 336 samples initially screened, 29 (8.6%) exhibited an inhibitory effect, and among them, 15 molecules (4.5%) decreased PHGDH activity by greater than 50%. A convergent pharmacophore approach contributed to the synthesis of *α*-ketothioamides by merging the arylketo moiety of 12-14 and the highlighted thioamide template after structural analyses (Figure 8D). Moreover, *α*-ketothioamides could selectively strain the proliferation of cancer cells with elevated PHGDH expression 76.

Qian Wang discovered novel non-NAD^+^ competing allosteric inhibitors for PHGDH using a structure-based design approach with the best IC50 of 28.1 ± 1.3 μM for enzyme inhibition. PKUMDL-WQ- 2101(Figure 8E) and PKUMDL-WQ-2201 (Figure 8F) were confirmed to specifically bind to PHGDH in PHGDH-amplified breast cancer cells with EC50 values less than 10 μM in serine-replete media. PKUMDL-WQ-2101 and PKUMDL-WQ-2201 inhibited PHGDH activity mainly by forming hydrogen-bond networks with R134, K57, and T59 of site I and T59, T56, and K57 of site II, respectively, which limits the movement of the rigid domains and prevents the active sites from closing, thus stabilizing PHGDH in the inactive conformation 77. Site I is close to the active site and the NAD+/NADH- cofactor-binding site, with a volume of 847 Å3 and a predicted maximal pKd of 8.71. It shares residues Gly 78, Val 79, Asp 80, Asn 81, and Val 82 with the active site. Site II is located in the substrate-binding domain, with a volume of 463 Å3 and a predicted maximal pKd of 7.79 78, 79. PKUMDL-WQ-2101 and PKUMDL-WQ-2201 also suppressed tumor growth in mice.

*Natural compounds.* Guo J et al. found that natural compounds inhibit the activity of PHGDH. Azacoccone E, the first natural PHGDH inhibitor discovered by screening an in-house database of NPs, had an optimal IC50 of 9.8 ± 4.3 μM (Figure 8G). Furthermore, azacoccone E was a non-competitive inhibitor in a time-dependent manner as identified by the observed enzyme inhibition kinetics. Molecular docking demonstrated that azacoccone E fit at the allosteric site of PHGDH, which is essential for diminishing enzyme activity. A ligand-binding pocket with two protruding arginines and dramatically hydrophilic hydrogen bindings of Arg118 with two phenol groups and Arg113 with lactam carbonyl was predicted. Azacoccone E selectively inhibited PHGDH-expressing cancer cell proliferation and showed dose-dependent proapoptotic activity in HeLa cells 80. However, the anti-tumor mechanism of azacoccone E remains unknown.

Zheng et al. extracted Ixocarpalactone A (Iox A) from dietary tomatillos (*Physalis ixocarpa*) and reported markedly inhibited PHGDH activity with an IC50 value of 1.66 ± 0.28 μM (Figure 8H). An MST assay and molecular docking experiments demonstrated that lox A directly coordinated at the allosteric site in the back side of the active site of PHGDH 81. The ligand-binding pocket was dramatically hydrophobic, and hydrophobic interactions between Val301 and ring A, Pro331 and ring D, and Ile333 and ring B were predicted. Several hydrogen bonds were predicted between Gln302 with the carbonyl group of ring A, Asp305 and Thr313 with the 3-hydroxyl group, and Pro327 with the 22-hydroxyl group. Iox A was a non-competitive inhibitor relative to the substrate of NAD co-enzyme, which decreased the potential toxicities. Iox A selectively suppressed the proliferation of high PHGDH-amplified cancer cell lines and dose-dependently promoted the apoptosis of cells in a micromolar concentration. Iox A markedly restrained the tumor growth in a xenograft mouse model with low toxic effects. Iox A was identified as a novel natural PHGDH inhibitor with high specificity and low toxicity for the treatment of pancreatic cancer.

## PHGDH and tumor resistance to chemotherapy drugs

Metabolic rewiring plays an essential role in the development of drug resistance 82-85. In triple-negative breast cancer (TNBC) cells exposed to doxorubicin, the glucose flux for serine synthesis was increased by upregulating PHGDH. Serine was then transformed into GSH, which antagonized the doxorubicin-induced formation of ROS. Consequently, inhibition of PHGDH via shRNA caused doxorubicin-induced oxidative stress and increased doxorubicin sensitivity. The enhancement of doxorubicin efficacy through simultaneous suppression of PHGDH was demonstrated in a mouse tumor model 86. In ER+ breast cancer, PHGDH may be a novel therapeutic target to reverse recurrence/resistance to tamoxifen therapy 87. In multiple myeloma (MM) cells, the higher activity of SSP contributed to bortezomib resistance caused by upregulated PHGDH and increased anti-oxidant capacity. Interestingly, PHGDH is upregulated in different BTZ-resistant MM cells and serine starvation improves the efficacy of BTZ 88.

High PHGDH also contributes to tumor resistance to targeted therapy. In response to vemurafenib treatments of a vemurafenib-resistant melanoma cell line, SK-MEL-28VR1 established from parental BRAF V600E SK-MEL-28 cells, PHGDH was elevated to support cell proliferation by supplying nucleotides. Knockdown of PHGDH or using methotrexate to inhibit nucleotide metabolism by impeding the folate cycle downstream of serine biosynthesis can sensitize SK-MEL-28VR1 cells to vemurafenib 89. The PHGDH level was also significantly increased in EGFR inhibitor erlotinib-resistant lung adenocarcinoma PC9ER4 and HCC827ER9 cells, which regulate the transcriptions of genes associated with DNA damage repair and nucleotide metabolism. Disturbing PHGDH by siRNA or NCT-503, an inhibitor of PHGDH, the tumoricidal effect was improved and sensitivity to erlotinib was restored in cell lines and xenografts 90, 91. Additionally, suppression of PHGDH can eradicate advanced or metastatic clear cell renal cell carcinoma (ccRCC) resistant to HIF2α antagonists by inducing apoptosis 92. PHGDH knocked down with RNAi and knocked out by CRISPR/Cas9 or inactivated by inhibitor can overcome tyrosine kinase inhibitor (TKI) drug resistance, including sorafenib, regorafenib, or lenvatinib, in hepatocellular carcinoma (HCC) 93. Taken together, the high expression of PHGDH is dramatically related to tumor resistance to chemotherapies, and treatment with PHGDH inhibitor works synergistically with chemotherapy drugs and may be an effective approach to improve overall patient survival.

## Conclusions

Targeting unique features of tumor metabolism is increasingly being deliberated as a successful approach. The strong dependence of cancer cells on certain nutrients or metabolites provides a therapeutic window and potentially has prognostic value. Although it has been more than 30 years since serine metabolism was proved to associated with tumorigenesis 31, the mechanism for this observation remains unclear. PHGDH is a rate-limiting enzyme in the serine biosynthetic pathway that consists of three enzymatic steps branching off the glycolytic pathway. Cells with high levels of PHGDH were connected with rapid proliferation, migration, and glycolytic metabolism supplying serine by SSP flux even when serine was available 33. It is important to identify at what time point PHGDH expression is high during tumorigenesis, how PHGDH promotes tumorigenesis and metastasis, and the relationship between PHGDH and tumor resistance.

Due to the physiology of the blood-brain barrier, the *de novo* synthesis of serine in the central nervous system (CNS) is necessary to supply amino acids required in the brain 94. To avoid potential neurological side effects, PHGDH inhibitor (or SSP enzyme inhibitor) must not cross the blood-brain barrier, obstructing serine homeostasis in the CNS. Many PHGDH inhibitors in the plasma are unstable and the anti-tumor mechanism of most parts of PHGDH remains unknown. Moreover, it is difficult to set thresholds to determine PHGDH-dependent and non-dependent tumors using existing techniques. Not only can serine be imported from the extracellular compartment via amino acid transporters and synthesized from glucose flux, but it can also be converted from glycine and glutamine. It may lead to resistance to PHGDH inhibitors by supplementing serine in other ways. Considering these factors, the targeted inhibition of PHGDH in tumor patients with high PHGDH expression will face significant challenges.

## Figures and Tables

**Table 1:** The role of overexpression PHGDH in cancer

Tumor types	Characteristics
Melanoma^4^	glucose metabolism improved, luminal organization and polarity disrupted, the viability of the inner, matrix-deprived cells to survive in an anchorage-independent fashion preserved, promoted oncogenesis
Estrogen receptor- negative breast 33	poor prognosis, decreased overall survival, higher tumor grade, and high expression of the proliferative markers proliferating cell nuclear antigen and Ki-67
Cervical^36^	large tumor, poor prognosis
Pancreatic^37^	advanced TNM stage, large tumor, higher tumor grade, rapid proliferation, migration and invasion
Colorectal^38^	advanced TNM stage, large tumor, tumor differentiation, and shorter overall survival time
Gastric^39^	histological type, tumor stage, and preoperative carcinoembryonic antigen
Lung adenocarcinoma^10^	high incidence, poor prognosis, rapid proliferation and migration
Leukemia^2^	high cell survival
Glioma^35^	PHGDH interacted with and stabilized FOXM1 at the protein level, promoting the proliferation, invasion and tumorigenicity of glioma cells
Breast cancer^49^, ^50^	high level of oncometabolite D-2-hydroxyglutarate (D-2HG)

## Abbreviations

3-PG: 3-phosphoglycerate
SSP: serine biosynthetic pathway
PHGDH: D-3-phosphoglycerate dehydrogenase
NADH: nicotinamide adenine dinucleotide
3-PHP: 3-phosphohydroxypyruvate
PSAT: phosphoserine amino transferase
TCA: tricarboxylic acid cycle
3-PS: 3-phosphoserine aminotransferase
PSPH: phosphoserine phosphatase
SHMT: serine hydroxymethyltransferase
THF: tetrahydrofolate
5,10-MTHF: 5,10-methylenetetrahydrofolate
GSH: glutathione
α-KG: α-ketoglutarate
Glu: glutamate
ORF: open reading frame
NF-Y: nuclear factor Y
ACT: aspartate kinase-chorismate mutase-tyrA
ASB: allosteric substrate binding
ER: estrogen receptor
TGF-β: transforming growth factor-β
ATF4: activating transcription factor 4
NRF2: nuclear factor erythroid-2-related factor 2
NSCLC: non-small cell lung cancer
mTORC: mammalian target of rapamycincomplex
SAM: S-adenosylmethionine
G9A: histone H3 lysine 9 methyltransferase
BTZ: bortezomib
EGFR: epidermal growth factor receptor
eIF4F: eukaryotic translation initiation factor 4F
FOXM1: forkhead box protein M1
MMP-2: matrix metalloproteinase 2
D-2HG: D-2-hydroxyglutarate
AML: acute myeloid leukemia
TNBC: triple-negative breast cancer
ROS: reactive oxygen species
MM: multiple myeloma
ccRCC: clear cell renal cell carcinoma
TKIs: tyrosine kinase inhibitors
HCC: hepatocellular carcinoma
CNS: central nervous system
